# Sounds of Strength: a programmatic evaluation of a music therapist led community-based program for Veterans in supportive housing

**DOI:** 10.3389/fpsyt.2025.1581291

**Published:** 2025-07-14

**Authors:** Lori F. Gooding, Flor del Cielo Hernandez, Diane G. Langston

**Affiliations:** Center for Music Research, Florida State University, Tallahassee, FL, United States

**Keywords:** music therapy, veterans, homelessness, community, military

## Abstract

**Introduction:**

Complexities such as TBI and co-occurring conditions such as homelessness impact Veterans’ awareness and use of health care services offered in the VHA and the community. Thus, targeted interventions that engage and reduce barriers to care among Veterans with interconnected challenges are needed. Research has suggested that music therapy interventions are well received by both active-duty Service Members and Veterans, but little is available on music therapy-based programming to address the complex needs of Veterans with interconnected challenges.

**Methods:**

The purpose of this project was to conduct a programmatic evaluation of a music therapist-led, community-based music program offered in a supportive housing complex to foster mental, emotional, and social health and wellbeing in Veterans encountering long-term and repeated homelessness, TBI, and other co-occurring conditions. We reviewed music therapist notes, attendance logs, participant demographic data, and unsolicited feedback from a 45-session music program grounded in a whole health and trauma-informed perspective to (a) identify the music interventions used and (b) explore how Veterans perceived the program. Demographic and attendance data were analyzed descriptively, and therapist notes and unsolicited feedback were examined thematically.

**Results:**

Results showed Veterans preferred rock, country, blues, and folk music, and singing and instrument play were the most common music strategies implemented in the program. Session topics most often included music, recreation/leisure, and emotions. The most common therapist objectives included self-expression, building rapport, and group cohesion. Both therapist notes and Veteran comments suggested that the program was appreciated, and it created a space where challenges could be addressed.

**Conclusion:**

This is one of the first music therapy programs presented in the literature to focus on Veterans experiencing homelessness and living in supportive housing. Results suggest that music therapy programming may be an innovative, well received option to engage Veterans with interconnected challenges. Results further suggest that sessions offered directly in supportive housing units and collaborating with Veterans on music planning may build trust and connection and empower them to take charge of their own health and wellbeing. Thus, music therapy-based programming may be uniquely positioned to address the needs of this marginalized population.

## 1 Background

The transition from active-duty military service to Veteran status is often a complex process not only for the Service Member, but for their family and support system as well. Stressors induced and exasperated by military service can place Veterans and their families at an increased risk for chronic health issues, often at a higher rate than their civilian counterparts (1). To address U.S. Veterans’ health and wellbeing needs, the Veterans Health Administration (VHA) provides medical care and social support services like readjustment counseling and outreach services for veterans (2). However, Veterans who use VHA services are a clinically complex group, and they can experience barriers to care (3). This is especially true for Veterans with interconnected challenges such as traumatic brain injuries (TBI) and co-occurring conditions like mental health conditions, substance use disorder, physical health conditions like pain, and social and transition issues like inadequate social support and homelessness (4). (*Note*: In alignment with language used by the U.S. Department of Veterans Affairs, the term ‘homeless’ is used throughout.)

To better address the complex needs of Veterans, the VHA has taken a whole health approach, moving away from a singular focus on disease-oriented care and empowering and equipping individuals to take charge of their health and wellbeing (5). As part of the whole health approach, the VHA now focuses on the full continuum of care, integrating non-clinical, wellbeing-oriented, and clinical services. Moreover, as Veterans have sought holistic and complementary treatment methods (6), the VHA has broadened its range of services to include evidence-based complementary/integrative approaches (7, 8).

Music therapy, an evidence-based integrative approach, has been recognized for its impact on military personnel across the clinic to community continuum (9). Music therapy services are currently offered across the VHA in group and individual format (10), both in person and via telehealth (11). Music therapy services for Veterans are provided to individuals with clinical, non-clinical, and wellbeing needs in a variety of settings including Veterans Affairs (VA) hospitals/clinics, VA Domiciliaries (12), permanent supportive housing, and VA living centers (13). Both active and receptive interventions like drumming (active) and song writing (receptive) have been used to improve functional outcomes (14), reduce stress and trauma (15), reduce isolation and increase socialization (16, 17), and promote community reintegration (15). Research has suggested that music therapy interventions are well received by both active-duty Service Members and Veterans (10), and there is emerging support for music-based interventions with Veterans with complex and interconnected needs (12). However, more research is needed to better understand how music therapy can support Veterans with interconnected challenges like mental health conditions, physical conditions, and homelessness.

The purpose of this project was to conduct a program evaluation of a music therapist-led, community-based music program offered in a supportive housing complex, designed to support and foster mental, emotional, and social health and wellbeing in Veterans encountering long-term and repeated homelessness, mental illness, and/or other health or transition issues. Our aim was to (a) identify the music interventions used in the program and (b) explore how Veterans perceived the music therapy interventions. To identify program characteristics, we explored the following questions as we evaluated the program:

- What types of Veterans were served in a music therapist-led, community-based music program?
- What music therapy informed interventions were used with Veterans who participated in a music therapist-led, community-based music program?
- Did Veteran participants prefer specific types of music, music interventions, or other elements implemented during the music therapist-led, community-based music program?
- What objectives were commonly addressed during the music therapist-led, community-based music program?

To explore the Veterans’ perceptions, we asked the following questions as we evaluated the program:

- How did Veterans perceive the music therapist-led, community-based music program?
- How did Veterans perceive their overall wellbeing while engaged in the music therapist-led, community-based music program?

## 2 Methods

### 2.1 Design

A descriptive, single unit, embedded case study design was used to evaluate the Sounds of Service music therapy program. We chose this observational, non-experimental design because it allowed us to evaluate Veterans’ experiences in music therapy within a real-life, single context using multiple sources of evidence (18, 19). This design also allowed us to maximize available resources while working within required privacy and confidentiality guidelines.

### 2.2 Participants

Veterans from all branches of the military ages 18 and older living in a U.S. Department of Housing and Urban Development-VA Supportive Housing ([HUD-VASH]; 20, 21) complex in a Northern Florida city were eligible to participate in the program. Veterans were eligible regardless of health status, time since transition out of active duty, VA service-connected eligibility status, or any demographic characteristic like sex or age.

### 2.3 Measurements and data analysis

Data were collected from two primary sources: the music therapist’s session notes and Veterans’ verbal and written program input. Attendance logs and music therapist session notes served as the primary sources of information. Attendance logs were used to track the total number of attendees per session. Session notes were used to collect data for each session on music genres used, and music therapy interventions implemented. Primary objectives targeted by the music therapist and topics addressed by the Veterans themselves were also tracked for each session. Additionally, the music therapist recorded narrative comments to address additional information not covered in the structured note. This included recording Veterans’ comments and other relevant clinical information.

To integrate the Veteran’s voice and input into the clinical program to the extent allowable within the required privacy and confidentiality guidelines, Veterans were asked to provide their military branch, preferred musical genres, and preferred ways to engage in music. This information was provided at the start of the program through a music use questionnaire; however, providing the information was voluntary and not required for participation in the program. Music use data were recorded in a spreadsheet and used to guide clinical programming. Veterans were asked to again provide their preferred music and preferred ways to engage in music at the end of the program. In addition to the music use data, narrative feedback was provided by two Veterans who chose to write notes in support of the program. These Veterans had verbalized support throughout the program and wanted to be sure their voices were included in the program evaluation.

All demographic and attendance data were analyzed descriptively to determine ranges, means, and standard deviations. Participants’ comments, taken from the therapist’s notes and Veterans’ feedback, were examined to determine if any common themes arose. To do this, we conducted a qualitative content analysis by coding and combining comments into thematic groups.

### 2.4 Theoretical conceptualization

Whole Health is a person-centered, evidence-informed approach that recognizes the relationship between the Veteran, their providers, and the community. Whole Health is rooted in the idea that engaging the whole person—physically, emotionally, mentally, and spiritually—is crucial to health and wellbeing (5). Whole Health aims to empower Veterans by offering personalized, proactive, patient-driven care guided by the Veteran’s values, goals, and vision for their own health and wellbeing (7). Currently, whole health is integrated in some VA sites; however, there have been calls for the Department of Veterans Affairs to scale the Whole Health system to fully transform care to be people-centered, comprehensive, and holistic (22). This would include providing care across a continuum and strengthening access to Complementary and Integrative Health (CIH) approaches like music therapy, which have been mandated as part of the standard VA medical benefits since 2017 (8).

In keeping with the Whole Health Model, we designed a group, music therapist-led music program to support and foster physical, mental, emotional, and social health and wellbeing among Veterans who successfully completed the HUDVASH program and engaged in music therapy treatment as part of their maintenance support. Our goal was to empower Veterans to take charge of their own health and wellbeing through Veteran-centered active and receptive music therapy interventions in a safe, familiar, and non-judgmental environment. We did this by focusing on whole person, personalized, proactive, Veteran driven care, and by including the Veterans themselves in constructing the structure of each session to foster collaboration with peers and the music therapist. We also focused on the core principles of trauma-informed care (safety, trustworthiness and transparency, peer support, collaboration and mutuality, empowerment, voice, and choice, and cultural, historical, and gender issues) (23), in part by integrating ongoing supervision by an experienced music therapist to ensure the music therapists leading sessions promoted ongoing safety, trust, and responsiveness to the Veterans.

### 2.5 Setting and program design

Sounds of Service was the title used for the duration of this program in 2019 and since has concluded. Sessions were offered in a HUD-VASH supportive housing complex located in a city in Northern Florida, USA. The housing complex provides housing for up to 50 homeless Veterans (24).

Sessions were led by music therapists from the music therapy private practice. One primary music therapist led the program, and in the event of the primary therapist’s absence, two additional music therapists served as substitutes. Additionally, a music therapy student was assigned to assist the board-certified music therapist during each session. The project was funded by the National Endowment for the Arts and the Florida Division of Cultural Affairs.

During the program, Veterans participated in active and receptive music therapy interventions including group music making (singing and group drumming), song discussion, lyric re-writing, and music-assisted relaxation. To promote trust and responsiveness, the Veterans also played an active role in determining the music experiences integrated in each session. Additional steps were taken to promote safety, trust, and responsiveness, including:

- The academic partner met with the VHA/HUD-VASH staff prior to the start of the program to ensure that the program’s design would be consistent with and responsive to the culture and needs of those who would be served. Communication continued throughout the project to support ongoing responsiveness to the Veterans’ needs and adapt to facility changes.
- The music therapy team actively consulted with HUD-VASH staff before, during, and after each session to promote Veteran safety.
- HUD-VASH/VHA staff worked with the music therapy team to ensure their policies and protocols were followed to facilitate Veterans’ safety and privacy.
- Sessions were offered directly in the HUD-VASH housing complex to increase accessibility for those without transportation. Sessions were also scheduled to maximize Veterans’ ability to participate.
- The MT-BC clinicians participated in regular supervision with the academic partner to address challenges and ensure ongoing responsiveness to the Veterans’ needs.

### 2.6 Session description

A basic session protocol was developed for structuring sessions. The protocol was intended to be a flexible guide rather than a directive session plan; as a result, each session was adapted by the music therapist based on who attended, the number of attendees, session location (location changed based on housing complex programming), and other factors. The session structure was also adapted based on input from and choices made by the Veterans. The protocol consisted of:

1. Introduction of therapist and participants.
2. Check in with participants. The music therapist and Veterans spent time discussing life events, changes since last time together, and other things Veterans chose to share.
3. Questions about music-related preferences and interventions. Veterans were asked about what music they had been listening to, instruments they wanted to play, music they wanted for the session, strategies they would like to participate in during the session, and so on.
4. Music therapist led re-creative music interventions: The music therapist led live singing and instrument play using preferred music options shared at the onset of the session or requested by Veterans during previous sessions. Re-creative interventions were selected because the Veterans “expressed their desire to play live music” (MT-BC session notes).
5. Veteran selected/led music interventions: Veterans selected and/or led music interventions like music listening, singing, and instrument play. Music selections included songs that had previously been introduced by the music therapist and/or music introduced by the Veterans. Veterans also brought their own recordings and/or instruments to the sessions.
6. Other music interventions: Other interventions were integrated when appropriate based on group and individual needs and requests from the Veterans. Interventions used included composition/songwriting, improvisation (primarily percussion based), and receptive interventions like song discussion.
7. Reflection and Feedback: Throughout the session, the music therapist created space for reflection, reminiscing, discussion, and feedback related to the music experiences during the session. Reflection questions were asked based on the music, group discussion, and comments shared by the Veterans. These questions were designed to elicit both Veterans’ input for future sessions and their insights and feelings. This approach allowed Veterans to shape future session content while also supporting emotional processing and personal reflection. Time was also spent summarizing the day’s experiences at the end of each session to provide closure before ending the session.

## 3 Results

### 3.1 Program characteristics

A total of 45 sessions occurred during the program. Of these, 42 were group music sessions, two were canvasing sessions, and one was a demonstration session. Canvasing sessions were focused on recruiting participants, and these occurred at the start of the program. Twenty people were engaged during the canvasing sessions, and a total of 21 unique individuals participated in the Sounds of Service program. Group size for the music sessions ranged from two to five individuals, with a cumulative total of 120 Veteran attendances. Veterans represented four branches of the U.S. military: the U.S. Army, U.S. Air Force, U.S. Marine Corps, and U.S. Navy. Among the participants, two Veterans identified as female, while the remaining individuals identified as male.

Veterans were asked, pre and post program, to indicate their preferred music therapy interventions. Live music listening and singing were identified as most preferred at both pre and post program. The music therapist also tracked music therapy interventions used in each session, and the most frequently utilized interventions included singing (82% of sessions), instrument play (69%), song discussion (53%), drumming (36%), and music listening (15%). Veterans were additionally asked to indicate their preferred music genres pre and post program; they indicated a preference for rock, country, blues, and folk music, with rock and country emerging as the most frequently utilized genres during sessions. Most frequently used genres were based on music therapist session notes. According to the pre and post data collected from the Veterans, musical preferences remained the same over the course of the program, and no reported changes were noted.

During each session, the music therapist tracked objectives. The three most frequently addressed objectives during the music therapy sessions were fostering self-expression (69%), building rapport (62%), and enhancing group cohesion (51%). Additional objectives addressed included mood enhancement (39%), developing leisure skills (29%), improving social skills (24%), reducing isolation (22%), and fostering coping skills (18%). Objectives such as self-regulation, stress reduction, and reminiscence were addressed at rates lower than 1%. **Table 1** presents the distribution of primary objectives addressed across sessions.

**Table 1:** Frequency of goals addressed during music therapy sessions in percentages.

Session objective	Objectives addressed in session
Self-Expression	69%
Building Rapport	62%
Group Cohesion	51%
Mood Alteration	38%
Leisure Skills Development	29%
Social Skills Development	24%
Decrease Isolation	22%
Coping Skills Development	18%
Self-Regulation	9%
Stress Reduction/Relaxation	7%
Reminiscence	2%
Closure	2%

Topics discussed during sessions were tracked by the music therapist, and the most often addressed topics related to music (84%), recreation and leisure (53%), emotions (51%), relationships (48%), and military service (46%). Other topics frequently discussed in sessions were coping, family, substance use disorder, finances and employment, and homelessness. Veteran verbal and musical engagement and group cohesion were assessed weekly by the music therapist using a standardized 5-point scale. To promote consistency across therapists, a shared rating guide was developed. Engagement was defined as observable participation in either verbal or musical activities during the session. Verbal engagement included behaviors such as responding to questions, sharing personal reflections, or contributing to group discussions. Musical engagement encompassed actively singing, playing instruments, improvising, or participating in music therapy interventions. Group cohesion was defined as the sense of connection, trust, and mutual support among group members, as demonstrated through active participation and positive interactions. Results showed verbal engagement (M = 4.2, SD = 1.0), musical engagement (M = 3.3, SD = 1.3), and group cohesion (M = 4.3, SD = 0.9).

### 3.2 Veterans’ comments

The authors conducted a qualitative content analysis on 30 comments shared by Veterans during sessions; these comments were obtained from session notes. Each comment was coded and combined into themes, which the team discussed and organized until agreement was reached. To enhance the trustworthiness of our thematic analysis, the authors conducted the coding process, and an additional researcher—who did not participate in the study—reviewed the session notes independently. Discrepancies were discussed and resolved through consensus to minimize bias.

Recurring keywords such as “together,” “therapy,” and “appreciate” were identified. Through open coding and collaborative discussions, six overarching themes emerged: Music (*n* =10), challenges (*n* = 6), daily life (*n* = 4), social connection and interaction (*n* = 4), appreciation and enjoyment (*n* = 4), therapeutic benefits (*n* = 2), and other (*n* = 3). **Table 2** depicts the comments grouped by key themes from the Veterans’ comments.

**Table 2:** Veteran’s comments grouped by themes.

Theme	Comments
Appreciation/Enjoyment	“I appreciate all you do for us. You do the work of ten people.” “I’ve enjoyed all we’ve done” “I love what we did today. I love being creative and making something that’s just ours.” “I love what you do here, and I really do get a lot out of it. I wish more of the guys would join.”
Challenges	“I had tea on the stove and bathroom business to attend to” “The government forced me into retirement by what was prophesized in the Bible long ago, where the mark of the beast shall appear and paper money will be taken away…” “Some people go to college and get degrees and then act like they’re better than everyone else. I didn’t go to college, but I’m not stupid.” “While I’ve loved coming to these, this is likely the last time.” “I know you do therapy things here but it’s play time for me and I can’t get into ‘work’ mode if I start my day with play.”
Daily life	“10 minutes I have” “Today is a good day. It’s beautiful outside.” “Life is still worth living even if it’s inconvenient–even when it’s traumatic.” “Life’s still good-still worth living.”
Music	“Glad to play together” “I’m glad they finally got someone back here to jam with us!” “Music like this transcends time and generation, and we have to keep it alive by playing it and talking about it.” “It shows that you really understand and relate to it, and then others will as well. That’s the magic of music.” “It sounds better than I thought it could.” “I never thought I could write a song” “Feeling the music,” is different from “just playing music,” “Music can take you to another place–remembering the good times.” “Because the music itself makes you feel good.” “Music group is a good time”
Other	“A very attractive woman who got all the guys to come to session” “I know you’ll be back. This isn’t it.” “See my girlfriends the nurses”
Social Connection and Interaction	“Glad to play together” “It gets us all together.” “Interaction” “I love what you do here, and I really do get a lot out of it. I wish more of the guys would join.”
Therapeutic Benefits	“No, man, this is therapy. This isn’t just fun, but it helps to soothe the mind, body, and soul.” “Even though it’s dark and rainy outside, I wanted to come to music and make my day better.”

### 3.3 Therapist notes

We followed the same process in analyzing 41 music therapist session notes as we did for the Veterans’ comments. Session notes were not recorded for the demonstration and canvasing sessions (*n*=3), and notes were missing for one session. This left a total of 41 notes for analysis. Our team identified four primary themes from the music therapist’s notes: (a) appreciation for the group (*n* = 13), (b) objectives and expectations for the group (*n* = 5), (c) challenges and barriers to attendance (*n* = 4), and (d) attendance (*n* = 4).

## 4 Discussion

The purpose of our study was to evaluate a music therapist-led, community-based music program offered in a supportive housing complex. This program was designed to support and foster mental, emotional, and social health and wellbeing in Veterans encountering long-term and repeated homelessness, mental illness, and/or other health or transition issues. To evaluate the program, we identified (a) Veterans served, (b) music therapy interventions implemented, (c) objectives addressed, and (d) Veterans’ perceptions of the program and their wellbeing as reported in music therapist session notes. Over the course of 45 sessions, we served 21 individuals from four branches of the military who largely identified as male. Singing and instrument play were the most common musical experiences, and rock and country were the most integrated musical genres. Per music therapist session notes, self-expression, building rapport, and group cohesion were the most addressed objectives. “Music” was the most discussed topic by the Veterans, and “appreciation and enjoyment” for the program was the most common topic identified by the music therapist. “Challenges and barriers” were identified by both the Veterans and the music therapist. Additional topics addressed included objectives (music therapist), social connection/interaction (Veterans), and daily life (Veterans).

Our findings illustrate the potential impact of music therapist-led music programming on Veterans, particularly in fostering the development of meaningful recreational/leisure skills while also providing a safe space—through music therapist-led experiences—for Veterans with complex needs to express themselves, build connection, and discuss challenges. Comments like “glad to play together,” “It shows that you really understand and relate to it, and then others will as well.” “The magic of music,” “It gets us all together,” and “I love what you do here, and I really do get a lot out of it. I wish more of the guys would join” highlight the Veterans’ perceptions that the program promoted the development of healthy leisure experiences, addressed social isolation, and promoted a sense of community (**Table 3**). Vetro-Kalseth et al. (25) found similar results in their work using a phased music therapy group to support Veterans’ reintegration. In that project, Veterans highlighted benefits of participating in music therapy groups like having skill-building opportunities, bolstering peer connections, decreasing isolation, enhancing camaraderie, and increasing social connections. Thus, engaging in music therapist-led group music therapy interventions appears to be a well received, promising option to address the needs of both Veterans transitioning (25) into the community and those living in the community with more expansive needs.

**Table 3:** Session notes grouped by themes. .

Theme	Comments
Appreciation for the group	Pt’s expressed appreciation of services and intent to return for next session. Pt’s expressed gratitude for services upon exit. Both pt’s expressed appreciation of services upon exit. Pt expressed gratitude for services upon exit. Pt’s expressed gratitude for services and excitement to continue sessions. Both pt’s stated they enjoyed hearing old rock songs ‘stripped down’ in sessions as it provided new insight into the music and new memories connected to the songs. Pt present expressed appreciation of services Both pts expressed gratitude for services upon exit. Pt expressed gratitude for services upon exit. Pt’s expressed gratitude for services and excitement to continue sessions. Pt’s expressed gratitude for services and excitement to continue sessions. Pt gave MT-BC a hug and verbal thanks for sessions. One client wrote a letter regarding his feelings towards MT sessions and desire for them to continue and dropped it off for administration.
Challenges and Barriers to Attendance	Pt did not attend due to ambulation problems. Community members expressed difficulty attending due to physical limitations. Community members expressed gratitude for our music program but also report that there is a general community suspicion/mistrust/misconception that we work for the state or the VA, and that we could get them in trouble. Patient stated he came to music for fun, not because he’s crazy, and “we all needed to clear” on that before session could proceed.
Goals and wants from group	The most mentioned goals within the community were 1) interaction, 2) positive recreation, 3) motivation/focus/inspiration, and 4) ‘Music with a meaning. Participants expressed desire to do more during sessions like field trips, attending concerts, etc. and expressed appreciation of services and desire to have services extend past July end date, ideally 2x/week. Pt expressed excitement for next session to write another song. Pt spoke at length of plans to buy the ‘fretless wonder’ Gibson guitar he’s wanted for forty years to ‘get serious about music. Pt expressed enthusiasm towards music therapy sessions, stating he spent most of his time alone in his apartment.
Attendance	Pt reports planning to return for future sessions. Pt’s expressed appreciation of services and intent to return for next session. Pt expressed appreciation of services upon exit and excitement to meet at next session. Pt expressed excitement for next session to write another song.

### 4.1 Considerations regarding program characteristics

Based on our results, it appears that the Veterans enjoyed engaging in and with music, mainly through active music making (e.g., singing, drumming) or through song discussion (verbal processing of the musical and verbal elements of a song). Previous research has shown that Veterans reported music listening as their most frequent leisure activity (26) and that Veterans have found opportunities to connect with others, build coping skills, and grow through music (27). Veterans who participated in the Sounds of Service program had similar responses, sharing “Glad to play together,” “I never thought I could write a song,” and “This isn’t just fun, but it helps to soothe the mind, body, and soul.” These comments suggest that the Veterans who participated in our project not only enjoyed the program, but also had opportunities to connect, build coping skills, and grow.

In designing our program, we intentionally tailored the program to meet the unique needs of those who would be participating. This included working collaboratively with the Veterans to determine what interventions would be integrated into the sessions. This appeared to be a positive approach, as illustrated by one Veteran who stated, “I love being creative and making something that’s just ours”. Their comment underscores the importance of tailored music therapy interventions and highlights the need to collaboratively engage Veterans in music programming.

Equally important, one Veteran shared “I’m glad they finally got someone back here to jam with us,” highlighting the importance and need to make therapy-based experiences accessible for Veterans, particularly those facing barriers to services. Offering Sounds of Service directly in the supportive housing unit appeared to support participation as well as create connections among the housing community residents (“It gets us all together”). Thus, it appears that group, music-therapy based programming should be structured to be (a) inclusive of both active (i.e., making) and receptive (i.e., responding to) music interventions, (b) collaborative and tailored to the Veteran participants, and (c) accessible. Additional research is needed to explore how specific interventions can better support Veterans facing complex and interconnected challenges such as Post Traumatic Stress Disorder (PTSD), depression, chronic pain, and homelessness.

### 4.2 Considerations regarding veterans’ and music therapist’s perception

Music therapy has been found to be well received by Veterans (10), potentially creating a path to address participants’ interconnected needs when more traditional approaches are viewed with distrust. According to Bradt et al. (28), music therapy may not be associated with the same stigma as other therapies, may be more accepted, and may lower barriers, particularly in terms of mental health care. Because music therapy services for Veterans emphasize evidence-based, person-centered interventions that promote the whole health of the individual (29), music therapy may increase accessibility and reduce barriers to participation when integrated into the holistic, “whole health” approach used to serve Veterans from the clinic to the community.

Trauma-informed care (TIC), which emphasizes safety, trustworthiness, peer support, collaboration, and empowerment (23), has been endorsed with military-connected populations (30). Music therapy naturally integrates these principles by creating a supportive environment that acknowledges the impact of trauma while fostering resiliency and recovery. By incorporating the TIC tenets along with the core principles of whole health, music therapists can provide emotional safety, encourage trust-building, and empower Veteran’s health journey (31, 32).

Veterans’ comments collected during the program highlighted the significant emotional and personal value participants placed on the music therapy sessions. Veterans expressed gratitude for the sessions and enthusiasm for future attendance, demonstrating strong engagement with the program. Participants also reported forming personal connections and gaining new insights through the experience. For example, some noted that hearing classic rock songs in a stripped-down format provided them with a fresh perspective and deeper emotional connection. This impact was further emphasized by two Veterans who wrote and delivered an unsolicited letter detailing the benefits of the music therapy program and underscoring the value of these sessions as a vital resource for Veterans. They also expressed a strong desire for a dedicated staff member to support and sustain the program. Additionally, participants’ goals for expanded activities and continued sessions further underscore the program’s importance in their lives, emphasizing the need for ongoing support and development.

Congruent with the Veteran’s perceptions expressed in the notes they wrote to support the program; music therapist’s session notes showed the Veterans’ appreciation and value for the services provided. According to these notes, participants articulated several goals for their engagement in music therapy, with the most common being social interaction, positive recreation, motivation, and finding personal meaning in music. Some participants expressed enthusiasm for expanding program activities, including field trips, attending concerts, and extending sessions beyond the initial timeframe, ideally increasing frequency to twice per week. Individual goals also emerged, such as writing new songs in future sessions and pursuing personal musical aspirations, such as purchasing a long-desired instrument to deepen musical practice. These responses underscore the program’s role in fostering both individual and group development, suggesting that expanding opportunities for engagement could further enhance participant outcomes. Future efforts should focus on sustaining and developing these services to meet the evolving needs of this community (33, 34, 35).

### 4.3 Other considerations for future programming

Financial, personal, and physical obstacles, confidence in the VA healthcare system, and privacy and security have all been identified as barriers to care among Veterans (36, 37). Veterans who participated in Sounds of Service expressed similar challenges and barriers to their participation. For example, physical challenges such as ambulation problems prevented some Veterans from attending. Additionally, community mistrust posed a barrier, as some individuals expressed concerns about the program’s affiliation with the state or the VA. There was also evidence of stigma surrounding therapy, with one participant clarifying they attended for enjoyment rather than because they were “crazy.” Comments like these highlight the need for increased accessibility and community education to address misconceptions and reduce barriers to participation.

## 5 Conclusion

Sounds of Service is one of the first music therapy programs presented in the literature to focus on homeless Veterans who are living in supportive housing. Our group-based, music therapist-led music program was designed from a whole health and trauma-informed perspective to address the unique needs of this marginalized population. We offered sessions directly in the housing complex to increase accessibility, and we collaborated with the Veterans, integrating their preferences and values to build trust and connection and empower them to take charge of their own health and wellbeing. Veterans appeared to both enjoy and benefit from the program, and the data collected suggest the potential of this type of program to meet the needs of Veterans with interconnected challenges.

We believe Sounds of Service is an important first step in expanding our understanding of music therapy with Veteran populations with complex, interconnected needs living in the community. It provides guidance on and a model for evidence-based clinical practice, academic research, and community connection. It also provides additional evidence on the benefits of music therapy treatment in supporting the clinic-to-community continuum. Future research and clinical learning are needed to explore the sustainability of programs such as this, but we hope this study demonstrates the feasibility of music therapy treatment in partnership with local community entities and a large health system.

## Data Availability

The datasets presented in this article are not readily available because data was not approved for public accessibility. Requests to access the datasets should be directed to LG, lgooding@fsu.edu.
